# Reading

**Published:** 1877-11

**Authors:** 


### READING.

PARENTS who do not exercise a careful supervision over the reading matter of their children, omit a duty of vital importance, and may reasonably anticipate subsequent disappointment, mortification, and sorrow, in the failure of those children to meet the expectations which had been formed of them. Aaron Burr revelled in the reading of infidel books in early youth; and yet, with talents to have made him a second Washington, he went down to his grave with the reputation of a corrupter of his kind, a traitor, and a murderer.

The son of the immortal John Howard, the friend of man, with all the advantages of a superior education and high social position, left to himself, to read what he listed—his mother being dead, and his father in foreign lands—fell into debauchery, and died a drunken madman, in the lunatic asylum of Leicester, before he was thirty-five.

It is recorded of the Emperor Paul, the Nero of modern times, one of the most execrable of men, if received histories are true, that he took the utmost delight in reading horrible tales of every description, in contemplating pictures of rapine, murder, and blood, only to practice them all when, a little later, he was placed on the throne of all the Russias.

Children can read and hear moral poison; and moral death is as certain to follow, as will physical death, if the poison of the apothecary is swallowed. A grain of strychnine is not the less fatal from being enveloped in sugar, or mingled with a hundred times its bulk in a teaspoonful of molasses.

A "splendid lecture" from a "splendid man," is rather the more pernicious, from having a single idea—atheistic, infidel, or impure; and a newspaper, a monthly, or a quarterly, may be, in the main, conducted with singular ability; but if a *roue*, or pantheist, or deist, have it under control, its very ability only adds to the malignancy of occasional propositions, in more or less direct consonance with the individual sentiments of the editor. We undertake to say, that this occasional jactatory exhibition of sentiments, radically subversive of moral purity and true religion, is a part and parcel of the design of too many of the monthly magazines and city newspapers, to some of which there is no responsible name—no distinct editor; while many of the various writers, taking advantage of their impersonality, shoot in the dark.

